# Hypertension and diabetes control along the HIV care cascade in rural South Africa

**DOI:** 10.1002/jia2.25213

**Published:** 2019-03-27

**Authors:** Jennifer Manne‐Goehler, Mark J Siedner, Livia Montana, Guy Harling, Pascal Geldsetzer, Julia Rohr, F Xavier Gómez‐Olivé, Alexander Goehler, Alisha Wade, Thomas Gaziano, Kathleen Kahn, Justine I Davies, Stephen Tollman, Till W Bärnighausen

**Affiliations:** ^1^ Division of Infectious Diseases Massachusetts General Hospital Harvard Medical School Boston MA USA; ^2^ Department of Global Health & Population Harvard T.H. Chan School of Public Health Boston MA USA; ^3^ Massachusetts General Hospital Harvard Medical School Boston MA USA; ^4^ Harvard Center for Population & Development Studies Harvard University Cambridge MA USA; ^5^ Africa Health Research Institute (AHRI) Mtubatuba South Africa; ^6^ Institute for Global Health University College London London UK; ^7^ Medical Research Council/Wits Rural Public Health & Health Transitions Research Unit School of Public Health University of the Witwatersrand Johannesburg South Africa; ^8^ INDEPTH Network Accra Ghana; ^9^ Department of Radiology, Brigham & Women's Hospital Harvard Medical School Boston MA USA; ^10^ Department of Cardiovascular Medicine Brigham & Women's Hospital Harvard Medical School Boston MA USA; ^11^ Center for Health Decision Science Harvard Medical School Boston MA USA; ^12^ Centre for Global Health King's College London London UK; ^13^ Institute of Public Health University of Heidelberg Heidelberg Germany

**Keywords:** ART, HIV care cascade, diabetes, hypertension, health systems

## Abstract

**Introduction:**

Participation in antiretroviral therapy (ART) programmes has been associated with greater utilization of care for hypertension and diabetes in rural South Africa. The objective of this study was to assess whether people living with HIV on ART with comorbid hypertension or diabetes also have improved chronic disease management indicators.

**Methods:**

The Health and Aging in Africa: a longitudinal study of an INDEPTH Community in South Africa (HAALSI) is a cohort of 5059 adults >40 years old. Enrollment took place between November 2014 and November 2015. The study collected population‐based data on demographics, healthcare utilization, height, weight, blood pressure (BP) and blood glucose as well as HIV infection, HIV‐1 RNA viral load (VL) and ART exposure. We used regression models to determine whether HIV care cascade stage (HIV‐negative, HIV+ /No ART, ART/Detected HIV VL, and ART/Undetectable VL) was associated with diagnosis or treatment of hypertension or diabetes, and systolic blood pressure and glucose among those with diagnosed hypertension or diabetes. ART use was measured from drug level testing on dried blood spots.

**Results and discussion:**

Compared to people without HIV, ART/Undetectable VL was associated with greater awareness of hypertension diagnosis (adjusted risk ratio (aRR) 1.18, 95% CI: 1.09 to 1.28) and treatment of hypertension (aRR 1.24, 95% CI: 1.10 to 1.41) among those who met hypertension diagnostic criteria. HIV care cascade stage was not significantly associated with awareness of diagnosis or treatment of diabetes. Among those with diagnosed hypertension or diabetes, ART/Undetectable VL was associated with lower mean systolic blood pressure (5.98 mm Hg, 95% CI: 9.65 to 2.32) and lower mean glucose (3.77 mmol/L, 95% CI: 6.85 to 0.69), compared to being HIV‐negative.

**Conclusions:**

Participants on ART with an undetectable VL had lower systolic blood pressure and blood glucose than the HIV‐negative participants. HIV treatment programmes may provide a platform for health systems strengthening for cardiometabolic disease.

## 1 Introduction

As HIV‐positive populations age in the era of widespread antiretroviral therapy (ART) availability, there is an urgent need to better understand the biological and health systems implications of the growing burden of cardiometabolic disease among people living with HIV (PLWH) 1, 2, 3. This is especially true for resource‐limited health systems in sub‐Saharan Africa that face large HIV epidemics and a growing prevalence of diabetes and hypertension among adults 4. A better understanding of the intersection between HIV and cardiometabolic conditions such as diabetes and hypertension offers a potential avenue for health systems strengthening, as care programmes that have been established for PLWH may provide a ready platform for the response to non‐communicable diseases. Leveraging the overlap between HIV and cardiometabolic care programmes, and aligning both with efforts to expand universal health coverage, has the promise of improving population health for all 5, 6.

Research at the nexus of these epidemics has been limited to date, in part due to scant sources of data that include information about both HIV and cardiometabolic conditions in the same participants 7. However, several studies have demonstrated both good feasibility and high levels of uptake when screening for multiple chronic diseases – including HIV, diabetes and hypertension – is implemented through an integrated health campaign 8, 9. Moreover, a recent study conducted in a rural South African cohort showed that participants in ART programmes have greater frequency of diagnosis and lifestyle counselling for both diabetes and hypertension as well as greater awareness of diagnosis and treatment for hypertension, compared to HIV‐negative participants 10. The objective of this study was to assess whether PLWH on ART with co‐morbid hypertension or diabetes also have improved chronic disease management indicators.

## 2 Methods

### 2.1 Data collection

This study was conducted using baseline data from Health and Aging in Africa: a longitudinal study of an INDEPTH Community in South Africa (HAALSI). HAALSI is a cohort of adults aged 40 and over based in the Agincourt sub‐district of rural South Africa 11, 12. Participants in HAALSI were randomly sampled from the Agincourt Health and Demographic Surveillance Site (HDSS). Enrollment took place from November 2014 to November 2015 13, 14. The baseline survey was administered by local fieldworkers and participant responses recorded in a Computer‐Assisted Personal Interview system 15. The study collected self‐reported demographic and economic characteristics as well as measures of healthcare utilization for HIV, diabetes and hypertension. Anthropometry, blood pressure, point‐of‐care glucose and dried blood spots (DBS) were also collected at the time of the survey. Participants provided consent for participation in the survey and dried blood spot collection.

Dried blood spots were tested for HIV using the Vironostika HIV 1/2 Ag/Ab MicroELISA System (BioMérieux, Marcy l'Etoile, France) and the Roche Cobas E411 Combi Ag (Indianapolis, IN, USA) respectively. We tested for HIV‐1 RNA using the BioMérieux NucliSens, which had a lower limit of detection of <100 copies/mL. All participants with a positive HIV antibody test also had a subsequent viral load (VL) test.

To ascertain whether patients with a positive HIV test were receiving ART, all participants with a positive HIV test were subsequently tested for exposure to emtricitabine (FTC) and lamivudine (3TC) using DBS. Testing was done at the University of Cape Town using high performance liquid chromatography which detected the presence of either drug at concentrations down to 0.02 μg/mL 16. This concentration detects use of either drug within the past 1.5 days.

### 2.2 Statistical analyses

Our primary predictor of interest is a categorical HIV status descriptor comprised of HIV serostatus, recent ART use and virologic suppression. Participants were defined as one of the following: (1) HIV‐negative; (2) HIV‐positive but not currently using ART (HIV‐positive/No ART); (3) HIV‐positive with recent ART use but with a detectable VL (ART/Detected VL); or (4) HIV‐positive participants with an undetectable VL (ART/Undetectable VL). Those participants who had an undetectable VL were considered to be in the ART/Undetectable VL even if their ART assay was negative.

Our outcomes of interest were as follows: (1) awareness of a hypertension diagnosis; (2) treatment of hypertension among those who meet criteria for diagnosis of hypertension (3) mean systolic blood pressure among those who meet criteria for a diagnosis of hypertension; (4) awareness of diabetes diagnosis; (5) treatment of diabetes among those who meet criteria for a diagnosis of diabetes and (6) blood glucose measurement among those who meet criteria for a diagnosis of diabetes. We defined a diagnosis of hypertension as any of the following: (1) a mean systolic blood pressure ≥140 mm Hg; (2) a mean diastolic blood pressure ≥90 mm Hg; (3) a self‐reported diagnosis of hypertension that had been made by a doctor, nurse or healthcare worker; or (4) self‐reported use of medication for hypertension prescribed by a doctor nurse or healthcare worker. We defined a diagnosis of diabetes as any of the following: (1) a fasting glucose ≥7.0 mmol/L; (2) a random plasma glucose ≥11.1 mmol/L; (3) a self‐reported diagnosis of diabetes that had been made by a doctor, nurse or healthcare worker; or (4) self‐reported use of medication for diabetes prescribed by a doctor, nurse or healthcare worker 13, 17, 18.

We used log‐binomial regression models to examine the association between diagnosis and treatment of hypertension or diabetes and stage in the HIV care cascade. These models were adjusted for age, sex, BMI, education and wealth quintile. We then fit linear regression models, also adjusted for age, sex, BMI, education and wealth quintile, to estimate differences in mean systolic blood pressure and blood glucose by stage in the HIV care cascade. We additionally adjusted the blood glucose model for fasting status. We then fit linear regression models including the entire population and second, excluding those with a BMI < 18.5, to minimize the possibility of confounding between HIV treatment and blood pressure or glucose due to advanced HIV disease stage. We used one‐way ANOVA to test for differences in means and chi‐squared tests to compare proportions. All statistical analyses were conducted in STATA v. 14 (College Station, TX, USA).

### 2.3 Ethics statement

This study received ethics approval from the University of Witwatersrand (#M141159), the Harvard T.H. Chan School of Public Health (#13‐1608), and the Mpumalanga Provincial Research and Ethics Committee.

## 3 Results and discussion

Our analytic sample included 4547 participants who had an HIV antibody test result and where applicable, ART exposure data available. Further details about participation in the HAALSI cohort are available elsewhere 10. The HIV‐negative participants were significantly older than the HIV‐positive participants. There were no differences in the sex distribution across groups but mean BMI and the percentage who were overweight or obese decreased from the HIV negative to the HIV positive/No ART and ART/Detected VL groups, then increased slightly in the ART/Undetectable VL group. (Table 1)

**Table 1:** Demographic and health characteristics across the HIV care cascade in the HAALSI cohort, 2015

	HIV negative	HIV positive/No ART	ART/detected VL	ART/undetected VL	*p*
Total	3512	301	183	551	—
Age (mean)	63.6	54.4	55.6	55.9	<0.001
Age 40 to 49	504 (14.4)	113 (37.5)	44 (24.0)	148 (26.9)	<0.001
Age 50 to 59	870 (24.8)	98 (32.6)	81 (44.3)	206 (37.4)	
Age 60 to 69	959 (27.3)	60 (19.9)	43 (23.5)	141 (25.6)	
Age 70 to 79	719 (20.5)	24 (8.0)	13 (7.1)	51 (9.3)	
Age 80+	460 (13.1)	6 (2.0)	2 (1.1)	5 (0.9)	
Sex (%)	1898 (54.0)	168 (55.8)	94 (51.4)	298 (54.1)	0.823
Education					<0.001
<1 year	1,660 (47.4)	130 (43.3)	70 (38.7)	226 (41.1)	
One to five years	1225 (35.0)	90 (30.0)	62 (34.3)	204 (37.1)	
Six to seven years	345 (9.8)	52 (17.3)	29 (16.0)	79 (14.4)	
Eight plus years	272 (7.8)	28 (9.4)	20 (11.0)	41 (7.4)	
BMI class (%)					<0.001
Underweight	160 (4.8)	21 (7.2)	17 (9.7)	37 (6.9)	
Normal	1133 (34.0)	129 (44.3)	83 (47.2)	235 (44.0)	
Overweight	971 (29.2)	68 (23.4)	44 (25.0)	145 (27.2)	
Obese	1064 (32.0)	73 (25.0)	32 (18.2)	117 (21.9)	
Wealth quintile					
Quintile 1	695 (19.8)	90 (29.9)	42 (23.0)	120 (21.8)	
Quintile 2	700 (19.9)	62 (20.6)	39 (21.3)	112 (20.3)	
Quintile 3	679 (19.3)	56 (18.6)	39 (21.3)	118 (21.4)	
Quintile 4	695 (19.8)	48 (16.0)	36 (19.7)	111 (20.2)	
Quintile 5	743 (21.2)	45 (15.0)	27 (14.8)	90 (16.3)	
Hypertension^a^ (%)	2394 (68.4)	143 (47.7)	84 (45.9)	255 (46.4)	<0.001
Diabetes^b^ (%)	446 (12.9)	21 (7.1)	11 (6.0)	48 (8.8)	<0.001

ART, antiretroviral therapy.

^a^Hypertension was defined as any of the following: (1) a mean systolic blood pressure ≥140 mm Hg or (2) a mean diastolic blood pressure ≥90 mm Hg or (3) a self‐reported diagnosis of hypertension that had been made by a doctor, nurse or healthcare worker or (4) self‐reported use of medication for hypertension prescribed by a doctor nurse or healthcare worker. ^b^Diabetes was defined as any of the following: (1) a fasting plasma glucose ≥7.0 mmol/L; (2) a random plasma glucose ≥11.1 mmol/L; (3) a self‐reported diagnosis of diabetes that had been made by a doctor, nurse or healthcare worker; or (4) self‐reported use of medication for diabetes prescribed by a doctor, nurse or healthcare worker.

In multivariable‐adjusted log binomial models, we found a significant relationship between being ART/Undetectable VL and awareness of hypertension diagnosis (adjusted risk ratio (aRR): 1.18, 95% CI: 1.09 to 1.28) and treatment of hypertension (aRR 1.24, 95% CI: 1.10 to 1.41) among those who met criteria for a diagnosis of hypertension, compared to those who were HIV negative. There were no significant relationships between stage in the HIV care cascade and receipt of diagnosis (aRR 1.14, 95% CI: 0.88 to 1.47) or treatment (aRR 1.04, 95% CI: 0.71 to 1.51) for diabetes (Tables S1 to S3).

**Figure 1:**
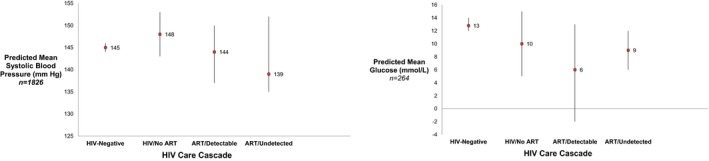
Predictive mean systolic blood pressure (BP) and glucose among those with diagnosed hypertension or diabetes by HIV Care Cascade Group in the HAALSI cohort, 2015

In multivariable‐adjusted linear regression models among those with diagnosed hypertension or diabetes, the ART/Undetectable VL group had a lower mean systolic BP (−5.98 mm Hg, 95% CI: −9.65 to −2.32) and lower mean glucose (−3.77 mmol/L, 95% CI: −6.85 to −0.69), compared to the HIV‐negative participants (see Figure 1). This effect was preserved when excluding those who were underweight, where being in the ART/Undetectable VL stage of the HIV care cascade was associated with a 5.94 mm Hg (95% CI: −9.68 to −2.20) decrease in blood pressure and 3.74 mmol/L (95% CI: −6.89 to −0.59) decrease in blood glucose as compared to the HIV‐negative group. The complete results of these regression analyses are provided in the Supporting Information (Table S1). We also provide results of a model of blood glucose restricted to only the non‐fasting population, in which our finding is preserved (Table S1).

In a community‐based study of individuals in rural South Africa, we found, among individuals with hypertension and diabetes, PLWH who had an undetectable VL were more likely to be aware of and treated for a comorbid diagnosis of hypertension, and, among those who were diagnosed with hypertension or diabetes, they also had lower mean blood pressure and blood glucose than their HIV‐negative counterparts. These findings are relevant to health systems in countries such as South Africa, which have large HIV epidemics and robust ART programmes, because it provides preliminary evidence that the HIV care platform may be leveraged to improve primary healthcare delivery for other chronic conditions 10. This finding is a critical extension of prior research in this cohort demonstrating greater self‐reported service utilization among those with well‐controlled HIV on ART, because it shows that chronic disease management indicators may also be improved among those with HIV who are also virally suppressed on ART and thus suggests that PLWH with comorbid cardiometabolic disease may fare better in the health system than their HIV‐negative counterparts. Further efforts to integrate care for HIV and other chronic diseases may offer an avenue to improve outcomes for all. For instance, in Uganda, several studies have demonstrated high rates of acceptance of community‐based integrated screening for HIV alongside diabetes, hypertension and malaria. In one study, they showed that as many as 95% of women and 52% of men were willing to participate in integrated campaigns 8, 9. In South Africa, integration of care is already beginning to be pursued through the Ideal Clinics initiative 19.

Although further data are required to establish the mechanisms for this relationship, one plausible hypothesis is that the greater frequency and intensity of contact afforded to ART users provides secondary health gains. For example, most people in HIV care see healthcare providers, counsellors, and pharmacists every two to three months, which may increases opportunities for diagnosis, health education, and treatment of other common comorbid diseases of HIV. Another possible explanation for this finding is that those who have an undetectable VL on ART have greater health literacy or are more likely to be adherent to health advice and medical therapies, and thus have better blood pressure and glycaemic control in the setting of comorbid hypertension or diabetes. Other alterative explanations may include HIV or ART‐specific effects on blood pressure or glucose, survivorship bias, or other unmeasured confounders. We attempted to mitigate the blood pressure and glucose lowering‐effects of advanced HIV reported elsewhere 20, 21, 22, 23 by conducting analyses restricted to people with a normal, overweight or obese BMI. Moreover, our population‐based study design additionally minimizes confounding by allowing inclusion of PLWH at all stages of the treatment cascade, as opposed to a clinic‐based population, which might bias estimation to those with health‐seeking behaviours.

There are several limitations to this study. First, we rely on cross‐sectional data, and thus cannot establish causality 24. This study is meant to advance hypothesis generation about the spillover effects of ART programmes for other chronic disease states. However, further investigation is needed to confirm these findings in longitudinal data and to test the effectiveness of various models of integrated care on both HIV and cardiometabolic disease outcomes over time. Second, our study had a small numbers of participants with diabetes, which limited our ability to make definitive conclusions about relationships between HIV and utilization of diabetes care. Third, we have a small sample size in the ART/Detected VL group so it is difficult to interpret whether ART programmes are also improving care for diabetes and hypertension in this strata of participants. Finally, there is the possibility of misclassification of ART use status as we assign the ART use categories primarily through an assay that detects a drug with a short half‐life. As such, it is possible that a participant may be classified as “not on ART” when they are on ART but have recently skipped doses. We attempt to overcome any misclassification of ART use status by including those who are virally suppressed but do not have ART detected in their blood in “ART/Undetectable” group.

## 4 Conclusion

In summary, this study supports a potential to harness ART programmes to strengthen systems of care for other HIV‐associated cardiometabolic conditions. More research is needed to assess the causal pathways underlying these relationships, and determine optimal models of care for integrated management of these conditions.

## Competing interests

The authors declare no conflicts of interest.

## Authors’ contributions

LM, JK, AW, TG and TB were involved in the data collection. JMG, MS, GH, PG, JD and TB designed the study. JMG conducted the analyses. All authors contributed to the writing and revision of the manuscript. All authors have read and approved the final manuscript.

## Supporting information


**Table S1**. Association between stage in the HIV care cascade and utilization of hypertension screening, diagnosis and systolic blood pressure among those with hypertension in the HAALSI cohort, 2015
**Table S2.** Association between stage in the HIV care cascade and utilization of diabetes screening, diagnosis and glucose among those with diabetes in the HAALSI cohort, 2015
**Table S3.** Diagnosis and Treatment of Hypertension and Diabetes by sex and HIV/ART status in the HAALSI cohort, 2015
