# Supplementary material for: Hypertension and diabetes control along the HIV care cascade in rural South Africa
Source: J Int AIDS Soc. 2019 Mar 27;22(3):e25213. doi: 10.1002/jia2.25213 (PMC6436499; doi:10.1002/jia2.25213)
Supplement: Supplementary file 1 — Table S1. Association between stage in the HIV care cascade and utilization of hypertension screening, diagnosis and systolic blood pressure among those with hypertension in the HAALSI cohort, 2015 Table S2. Association between stage in the HIV care cascade and utilization of diabetes screening, diagnosis and glucose among those with diabetes in the HAALSI cohort, 2015 Table S3. Diagnosis and Treatment of Hypertension and Diabetes by sex and HIV/ART status in the HAALSI cohort, 2015 [file JIA2-22-e25213-s001.docx]

**Appendix Table 1. Association between stage in the HIV care cascade and utilization of hypertension screening, diagnosis and systolic blood pressure among those with hypertension in the HAALSI cohort, 2015**

|  | **Diagnosed HTN** | **Treated HTN** | **Systolic BP (mm Hg)** | **Systolic BP (mm Hg)** |
| --- | --- | --- | --- | --- |
| Covariates | **aRR + 95% CI** | **aRR + 95% CI** | **95% CI^1^** | **95% CI^2^** |
| Age 50-59 | 1.05 | 1.31*** | 4.75** | 4.90** |
|  | (0.95 - 1.16) | (1.08 - 1.60) | (0.84 - 8.66) | (0.945 - 8.85) |
| Age 60-69 | 1.14*** | 1.66*** | 0.75 | 0.64 |
|  | (1.03 - 1.26) | (1.37 - 2.01) | (-3.29 - 4.79) | (-3.444 - 4.73) |
| Age 70-79 | 1.29*** | 1.91*** | 1.17 | 1.17 |
|  | (1.17 - 1.44) | (1.57 - 2.36) | (-3.11 - 5.45) | (-3.17- 5.51) |
| Age 80+ | 1.26*** | 1.87*** | 5.27** | 4.94* |
|  | (1.12 - 1.42) | (1.51 - 2.31) | (0.41 - 10.13) | (-0.01 - 9.90) |
| Sex | 1.18*** | 1.23*** | -4.41*** | -4.78*** |
|  | (1.12 - 1.25) | (1.13 - 1.34) | (-6.74 - -2.07) | (-7.16 - -2.40) |
| Overweight | 1.14*** | 1.23*** | 4.36*** | 3.98*** |
|  | (1.07 - 1.22) | (1.10 - 1.37) | (1.58 - 7.14) | (1.12 - 6.85) |
| Obese | 1.24*** | 1.42*** | 7.34*** | 7.02*** |
|  | (1.16 - 1.32) | (1.28 - 1.57) | (4.56 - 10.12) | (4.16 - 9.88) |
| Education 1-7 | 1.06* | 0.98 | -0.21 | -0.16 |
|  | (0.99 - 1.12) | (0.90 - 1.07) | (-2.70 - 2.28) | (-2.69 - 2.38) |
| Education 8-11 | 0.96 | 0.91 | -3.95* | -3.92* |
|  | (0.87 - 1.06) | (0.78 - 1.07) | (-8.01 - 0.12) | (-8.02 - 0.19) |
| Education 12+ | 0.94 | 0.79** | -1.45 | -1.61 |
|  | (0.83 - 1.07) | (0.64 - 0.99) | (-6.57 - 3.66) | (-6.75 - 3.54) |
| Wealth Quintile 2 | 1.01 | 0.99 | 0.86 | 1.52 |
|  | (0.93 - 1.108) | (0.87 - 1.15) | (-2.76 - 4.47) | (-2.20 - 5.24) |
| Wealth Quintile 3 | 1.11** | 1.09 | -1.25 | -0.84 |
|  | (1.02 - 1.21) | (0.96 - 1.25) | (-4.82 - 2.32) | (-4.50 - 2.81) |
| Wealth Quintile 4 | 1.11** | 1.16** | -2.55 | -2.015 |
|  | (1.02 - 1.21) | (1.02 - 1.32) | (-6.06 - 0.96) | (-5.59 - 1.56) |
| Wealth Quintile 5 | 1.18*** | 1.31*** | -0.88 | -0.58 |
|  | (1.08 - 1.28) | (1.15 - 1.49) | (-4.54 - 2.77) | (-4.31 - 3.15) |
| **HIV+/No ART** | **1.06** | **0.85** | **3.43** | **4.33** |
|  | **(0.94 - 1.19)** | **(0.67 - 1.08)** | **(-1.62 - 8.48)** | **(-0.87 - 9.53)** |
| **ART/Detected VL** | **1.07** | **0.91** | **-1.19** | **-1.36** |
|  | **(0.91 - 1.26)** | **(0.67 - 1.24)** | **(-7.87 - 5.49)** | **(-8.178 - 5.47)** |
| **ART/Undetected VL** | **1.18***** | **1.24***** | **-5.98***** | **-5.94***** |
|  | **(1.09 - 1.28)** | **(1.10 - 1.41)** | **(-9.65 - -2.32)** | **(-9.68 - -2.20)** |
|  |  |  |  |  |
| Observations | 2,717 | 2,718 | 1,826 | 1,767 |

***^1^****Model with all participants with diagnosed hypertension;^2^Model restricted to participants with diagnosed hypertension and BMI>18.5, **p<0.05, ***p<0.01*

**Appendix Table 2. Association between stage in the HIV care cascade and utilization of diabetes screening, diagnosis and glucose among those with diabetes in the HAALSI cohort, 2015**

|  | **Diagnosed DM** | **Treated DM** | **Glucose (mmol/L)** | **Glucose (mmol/L)** | **Glucose (mmol/L)** |
| --- | --- | --- | --- | --- | --- |
| Covariates | **aRR + 95% CI** | **aRR + 95% CI** | **95% CI^1^** | **95% CI^3^** | **95% CI^3^** |
| Age 50-59 | 1.42* | 1.62* | -1.82 | -1.73 | -3.15 |
|  | (0.95 - 2.13) | (0.93 - 2.83) | (-5.76 - 2.12) | (-5.68 - 2.21) | (-7.92 - 1.63) |
| Age 60-69 | 1.49* | 1.45 | -3.57* | -3.38* | -4.99* |
|  | (0.99 - 2.25) | (0.82 - 2.58) | (-7.48 - 0.35) | (-7.32 - 0.55) | (-9.78 - -0.21) |
| Age 70-79 | 1.54** | 1.43 | -3.61* | -3.41 | -4.94 |
|  | (1.01 - 2.36) | (0.784 - 2.61) | (-7.72 - 0.51) | (-7.56 - 0.74) | (-9.95 - 0.06) |
| Age 80+ | 1.55* | 1.461 | -4.30* | -3.85 | -5.57 |
|  | (0.98 - 2.46) | (0.758 - 2.818) | (-9.05 - 0.46) | (-8.65 - 0.96) | (-11.31 - 0.16) |
| Sex | 1.04 | 1.025 | 1.20 | 1.30 | 0.83 |
|  | (0.87 - 1.23) | (0.804 - 1.306) | (-0.68 - 3.09) | (-0.59 - 3.20) | (-1.37 - 3.03) |
| Overweight | 1.00 | 1.137 | -0.18 | -0.50 | -0.31 |
|  | (0.81 - 1.24) | (0.826 - 1.564) | (-2.66 - 2.29) | (-3.02 - 2.02) | (-3.21 - 2.60) |
| Obese | 0.97 | 1.174 | -1.02 | -1.30 | -0.74 |
|  | (0.785 - 1.187) | (0.864 - 1.597) | (-3.39 - 1.35) | (-3.70 - 1.10) | (-3.58 - 2.10) |
| Fasting Status | --- | --- | -0.09 | -2.13* | --- |
|  | --- | --- | (-2.21 - 2.02) | (-4.43 - 0.18) | --- |
| Education 1-7 | 1.008 | 1.05 | -0.26 | -0.18 | -0.52 |
|  | (0.840 - 1.210) | (0.82 - 1.34) | (-3.84 - 3.33) | (-2.31 - 1.95) | (-2.97 - 1.92) |
| Education 8-11 | 1.109 | 1.067 | -0.592 | -0.22 | -0.81 |
|  | (0.823 - 1.496) | (0.69 - 1.64) | (-4.36 - 3.17) | (-3.81 - 3.376) | (-4.94 - 3.32) |
| Education 12+ | 1.297* | 1.23 | -1.46 | -0.63 | -0.76 |
|  | (0.964 - 1.744) | (0.81 - 1.86) | (-4.98 - 2.05) | (-4.41 - 3.15) | (-5.07 - 3.55) |
| Wealth Quintile 2 | 0.884 | 0.93 | -2.42 | -2.02 | -1.71 |
|  | (0.636 - 1.228) | (0.62 - 1.41) | (-5.71 - 0.87) | (-5.66 - 1.62) | (-5.76 - 2.34) |
| Wealth Quintile 3 | 0.987 | 0.95 | -2.21 | -2.95* | -2.64* |
|  | (0.727 - 1.339) | (0.64 - 1.41) | (-5.41 - 0.99) | (-6.32 - 0.41) | (-6.38 - 1.10) |
| Wealth Quintile 4 | 1.035 | 0.877 | -1.70 | -2.76* | -2.60* |
|  | (0.78 - 1.379) | (0.596 - 1.290) | (-5.14 - 1.74) | (-6.05 - 0.53) | (-6.27 - 1.07) |
| Wealth Quintile 5 | 1.147 | 1.025 | -2.19* | -2.17 | -1.46 |
|  | (0.86 - 1.53) | (0.707 - 1.49) | (-4.46 - 0.08) | (-5.68 - 1.33) | (-5.46 - 2.54) |
| **HIV+/No ART** | 1.030 | 0.920 | **-2.80** | **-2.08** | **-2.16** |
|  | (0.678 - 1.56) | (0.489 - 1.73) | **(-7.69 - 2.09)** | **(-7.29 - 3.13)** | **(-7.65 - 3.33)** |
| **ART/Detected VL** | 0.897 | 0.580 | **-7.13*** | **-7.14*** | **-8.17*** |
|  | (0.48 - 1.66) | (0.173 - 1.94) | **(-14.53 - 0.27)** | **(-14.56 - 0.27)** | **(-18.95 - 2.61)** |
| **ART/Undetected VL** | 1.138 | 1.037 | **-3.77**** | **-3.74**** | **-3.82**** |
|  | (0.88 - 1.469) | (0.712 - 1.51) | **(-6.85 - -0.69)** | **(-6.89 - -0.59)** | **(-7.40 - -0.24)** |
|  |  |  |  |  |  |
| Observations | 488 | 488 | 264 | 261 | 215 |

***^1^****Model with all participants with diagnosed diabetes;^2^Model restricted to participants with diagnosed diabetes and BMI>18.5, ^3^Model restricted to participants with random glucose only, **p<0.05, ***p<0.01*

**Appendix Table 3. Diagnosis and Treatment of Hypertension and Diabetes by sex and HIV/ART status in the HAALSI cohort, 2015**

|  | Men | Women | HIV-negative | HIV+/No ART | ART/Detected | ART/Undetected |
| --- | --- | --- | --- | --- | --- | --- |
| Total HTN* | 1227/2095 (58.6) | 1670/2471 (67.6) | 2394/3499 (68.4) | 143/300 (47.7) | 84/183 (45.9) | 255/550 (46.4) |
| Diagnosed HTN* | 739/1227 (60.3) | 1227/1670 (73.5) | 1617/2394 (67.6) | 90/143 (62.9) | 50/84 (65.5) | 189/255 (74.1) |
| Treated HTN | 475/1227 (38.7) | 853/1670 (51.1) | 1111/2394 (46.4) | 46/143 (32.2) | 32/84 (38.1) | 128/255 (50.2) |
| Total DM** | 223/2075 (10.8) | 307/2447 (12.6) | 446/3466 (12.9) | 21/296 (7.1) | 11/182 (6.0) | 48/546 (8.8) |
| Diagnosed DM** | 134/223 (60.1) | 178/307 (58.0) | 263/446 (59.0) | 11/21 (52.4) | 6/11 (54.6) | 29/48 (60.4) |
| Treated DM | 96/22 (43.1) | 132/307 (43.0) | 195/446 (43.7) | 7/21 (33.3) | 4/11 (36.7) | 20/48 (43.0) |

**HTN – hypertension, **DM – diabetes mellitus*
